# Investigating attentional control sets: Evidence for the compilation of multi-feature control sets

**DOI:** 10.3758/s13414-022-02566-4

**Published:** 2022-10-13

**Authors:** Simon Merz, Frank Beege, Lars-Michael Schöpper, Charles Spence, Christian Frings

**Affiliations:** 1 Department of Cognitive Psychology, Trier University, D-54286 Trier, Germany; 2 SAP Deutschland, 69190 Walldorf, Germany; 3 Department of Experimental Psychology, Crossmodal Research Laboratory, University of Oxford, Oxford, UK

**Keywords:** Vision, Attention, Contingent capture, Top-down control, Attentional capture, Multiple selection features

## Abstract

Top-down control over stimulus-driven attentional capture, as postulated by the contingent capture hypothesis, has been a topic of lively scientific debate for a number of years now. According to the latter hypothesis, a stimulus has to match the feature of a top-down established control set in order to be selected automatically. Today, research on the topic of contingent capture has focused mostly on the manipulation of only a single feature separating the target from the distractors (the *selection feature*). The research presented here examined the compilation of top-down attentional control sets having *multiple selection features*. We report three experiments in which the feature overlap between the distractor and the top-down sets was manipulated on different perceptual features (e.g., colour, orientation and location). Distractors could match three, two or one of the features of the top-down sets. In line with our hypotheses, the strength of the distractor interference effects decreased linearly as the feature overlap between the distractor and the participants’ top-down sets decreased. These results therefore suggest a decline in the efficiency with which distractors involuntarily capture attention as the target-similarity decreases. The data support the idea of multi-feature attentional control sets and are discussed in light of prominent contemporary theories of visual attention.

## Introduction

Nowadays, it is well established that people can attend to an object voluntarily, that is, on the basis of their behavioural goals, but also involuntarily, that is, in a stimulus-driven manner, as a result of the attention-capturing properties of environmental stimuli. The extent to which our behavioural goals affect stimulus-driven selection continues to constitute a lively topic of debate amongst researchers (see Folk et al., 1992; Goodhew et al., 2014; but see also Theeuwes, 2010). Many studies have examined stimulus-driven attentional capture using the peripheral (exogenous) cuing task, a paradigm assumed to measure stimulus-driven attentional capture (for the original task, see Posner, 1980; but see also Jonides, 1981; Jonides & Yantis, 1988; and see Büsel et al., 2020, for a recent meta-analysis). Yet, Folk and his colleagues (1992) have argued that the potential of a cue to involuntarily capture a participant’s attention is contingent on their current behavioural goals. Attentional control sets (top-down sets) are widely accepted as being a powerful factor when it comes to attentional capture (see Lamy & Kristjánsson, 2013; but see also Awh et al., 2012). However, the conditions under which top-down control sets counter attentional capture are still an area of intensive research (see Becker et al., 2013; Du et al., 2014; Goller & Ansorge, 2015; Kiss et al., 2013; Weichselbaum & Ansorge, 2018; Wyble et al., 2013; for a recent meta-analysis, see Büsel et al., 2020).

In their original study, Folk et al. (1992) presented a salient peripheral cue that was thought to automatically attract the participant’s spatial attention to its location. In order to test for the *contingent capture* of their participants’ attention, two different target conditions were introduced: a colour singleton target condition and an onset singleton condition. In the former condition (see Pashler, 1988, for the ‘singleton’ label, describing discrepant items), the participants had to identify a colour singleton (e.g., a red ‘X’ or ‘=’) presented together with three distractors (e.g., three green ‘X’s or ‘=’s) in the target display. In the onset singleton condition, the participants had to identify the shape of an onset singleton (i.e., the only stimulus that appeared randomly from any one of the four possible target locations) in the target display. The idea here was that the participants would apply a specific search template (attentional control set) according to the task requirements, to identify either a colour singleton or an onset singleton. In both (target) conditions, the target display was preceded by a cue display. The cue was either a colour singleton or an onset singleton (but did not include any information about the participants’ task of identifying either an ‘X’ or an ‘=’). In the valid trials, the cue was presented at the same location as the subsequent target stimulus. A trial was invalid when the cue was presented at a different location than the target. Intriguingly, cuing effects (i.e., faster responses in valid as compared to invalid trials) were only documented when the cues matched the participant’s current search templates. That is, when the participants were searching for a colour singleton as the target stimulus, only the colour singleton cues resulted in cuing effects, whereas the onset singleton did not give rise to any cuing effects (and vice versa). The authors interpreted these results in terms of the contingent capture of spatial attention. In order to become automatically selected (i.e., to automatically attract attention), a stimulus has to match the crucial feature that the participants are currently using as their search template (see also Gibson & Kelsey, 1998, for a display-wide account of contingent capture).

Evidence for a contingent capture of spatial attention has been provided with various features, such as colour (Ansorge & Becker, 2014; Ansorge & Heumann, 2003, 2004), size (Becker, 2010), abrupt onset (Folk & Remington, 1998; Pratt & McAuliffe, 2002), motion (Abrams & Christ, 2003; Folk et al., 1994), and even additional tactile (Mast et al., 2015) and auditory stimulation (Mast et al., 2017, for related cross-sensory discussions, see Spence et al., 2001). Furthermore, studies have revealed that participants might even set up multiple top-down sets in parallel (see Adamo et al., 2008; Adamo et al., 2010). The importance of contingent capture has been further underpinned by various studies using different paradigms, such as, for example, *temporal order judgement* tasks (see Born et al., 2015), *additional singleton tasks* (see Bacon & Egeth, 1994), or the *attentional blink paradigm* (see Folk et al., 2002). In addition to behavioural measures, the contingent capture hypothesis has also been supported by those studies that have used event-related potentials (ERPs) as the dependent measure (see Ansorge et al., 2011; Eimer et al., 2009; Kiss et al., 2013). Note that contingent capture has even been observed in those studies involving a non-spatial task (for a non-spatial *attentional blink* task, see Folk et al., 2008; for a non-spatial compatibility task, see Mast & Frings, 2014; Mast et al., 2015).

To date, there is a lot of evidence that multiple features of the target can be used to set up attentional control sets (e.g., Biderman et al., 2017; Büsel et al., 2018; Olivers et al., 2011). Yet, there is still the debate as to whether multiple features can be incorporated in one template (e.g., Büsel et al., 2018; Mast & Frings, 2014), or if multiple templates for multiple features are compiled and rapidly switched (e.g., Irons et al., 2012; Kerzel & Witzel, 2019; Roper & Vecera, 2012). Mast and Frings (2014) proposed an extension of contingent capture according to which top-down sets can be compiled with multiple features that serve different purposes. In order for a feature to become incorporated into the top-down set, it needs to be relevant for the execution of the current task. That could be by indicating the correct response (a *response feature*; e.g., shape, ‘x’ vs. ‘=’, as in Folk et al., 1992) or by a feature being used to separate the target from the distractors (a *selection feature*; e.g., colour in Folk et al., 1992). Therefore, the number of features that are implemented into the top-down set varies as a function of the task requirements. In their study, Mast and Frings demonstrated that whether an additional selection feature (in addition to the response feature) will become implemented into the top-down sets depends on whether that feature could be used to separate the target from distractors during the experiment. The central empirical prediction from Mast and Frings’ study is that the strength of attentional capture varies as a function of the feature overlap between the distractors and the participants’ current top-down sets (i.e., the target-distractor similarity; see also Ansorge & Heumann, 2003, 2004). This notion was subsequently linked to the idea of curiosity exploration (Frings et al., 2019).

Research on the topic of contingent capture has primarily focused on the manipulation of a single *selection feature* that could be used to separate the target from the distractor(s) (see Kiss et al., 2013, for an exception). However, one might easily imagine situations in which the processing of multiple features in a single top-down control set might be beneficial or even necessary in order to execute a task correctly (e.g., to separate traffic lights from digital advertisements; both might have an abrupt onset and even similar colours, but likely differ in terms of their shape and size; for a discussion on how the present results align with current developments in the action control literature, see the *General discussion* section). Such multi-feature attentional control sets might be expected to affect attentional capture in two potential ways. On the one hand, it could be argued that a distractor has to match all of the features of the current attentional control set in order to be selected automatically. Therefore, contingent capture should be driven by feature conjunctions. On the other, one might assume a binary comparison between the features of a stimulus and the current attentional control sets. Thus, a stimulus might have to match any of the features incorporated into the participants’ top-down sets in order to be selected. In line with Mast and Frings (2014), a binary compilation of multi-feature attentional control sets is hypothesized according to which the feature overlap between the top-down set and the distractor determines the strength of the attentional capture effects. If this were to be the case, the multi-feature approach could perhaps be understood as a more general extension of the contingent capture hypothesis with *multiple* selection and response features.

## Overview

**Fig. 1:**
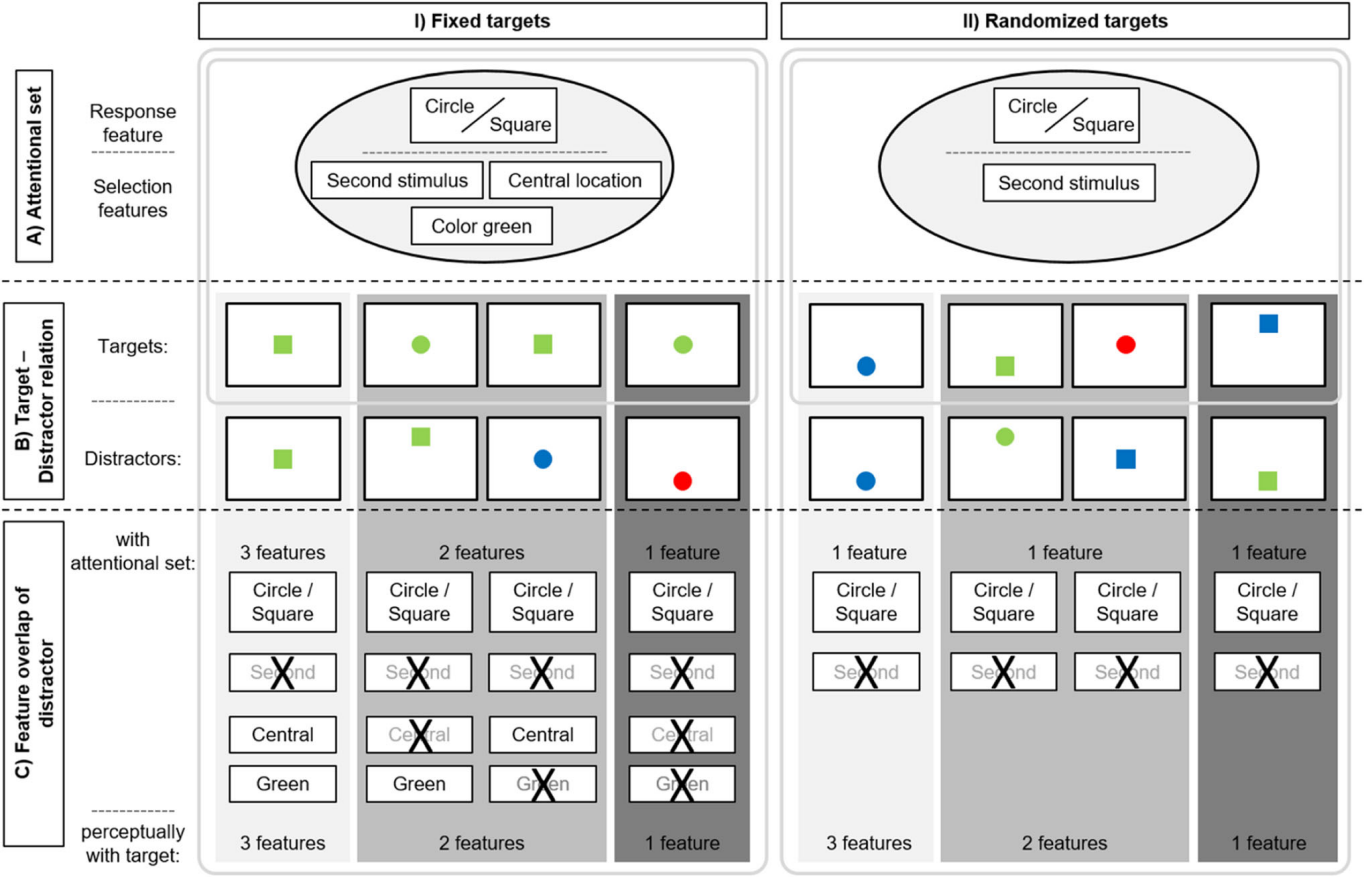
The figure depicts the proposal of multi-feature attention control sets as well as the experimental design used in the present manuscript. On the left, the fixed target condition is presented (I); on the right, the randomised target condition is presented (II). For the two different target conditions, different attention control sets are predicted. In both attentional control sets, the response feature shape (circle or square) as well as timing (second stimulus) were included to be able to conduct the task. **a** For the fixed target condition, the location (central) and the color (green) of the target was fixed and predictable and were therefore included into the attention control set. As for the randomized target condition, the location (centrally, above or below the centre) and the color (green, red and blue) were not fixed, therefore these features were not included in the attentional control set. Possible Target and Distractor combinations (some examples, non-exhaustive) are presented in the middle (**b**). At the bottom (**c**), the featural overlap between the distractor and either the attentional control set (upper) or the perceptual feature overlap between the distractor and target (lower) are presented. This illustrates the experimental logic of the present study – whereas the perceptual feature overlap is comparable for both target conditions, the feature overlap with the attentional set is different for both target conditions. For more information, see main text

In the present study, the participants’ ability to incorporate multiple selection features into their top-down sets was examined. As in previous studies (Mast et al., 2015; Mast & Frings, 2014), a non-spatial response compatibility task was used. During each trial, two stimuli were presented in close temporal succession. However, in order to prevent the participants from allocating their attention to a specific point in time (see Nobre, 2001; Nobre & Coull, 2010), the stimulus onset asynchrony (SOA) between the appearance of the distractor and the target was varied randomly on a trial-by-trial basis (80, 120 or 160 ms). Across all of the experiments in this study, the participants responded to the shape (square vs. circle; shape as the *response feature*) of the second stimulus (the target) while trying to ignore the shape of the first stimulus (the distractor). In the compatible trials, the target and the distractor were mapped on to the same response. In the incompatible trials, by contrast, the two stimuli were mapped on to opposing responses instead. The shape of the distractor stimulus was not correlated with the shape of the upcoming target stimulus (i.e., 50% of the trials were compatible) to prevent the participants from predicting the target by the distractor’s response feature. Beside shape being the response feature for all the experiments, the temporal feature of the target stimulus was the first selection feature in the attentional control set. That is, participants were tasked to respond to the second stimulus (of two), therefore the temporal feature allowed participants to clearly select the target against the distractor. This was constant throughout all experimental conditions and experiments, as visualized in Fig. 1.

In order to experimentally test whether multiple selection features were incorporated into the top-down set, an experimental environment was designed in which the target was predictable on at least two further stimulus feature dimensions (*fixed target* condition; in Experiment 1: location and colour; in Experiment 2: orientation and colour; in Experiment 3: location, orientation and colour). For example, the target was always presented in green and centrally on the screen in Experiment 1 (for a visualisation, see Fig. 1). This allowed the participants to incorporate the two additional features (location and colour) into the top-down set in order to select the target against the distractor as the distractor varied regarding these additional features (e.g., was presented in the centre as well as in the periphery; was presented in green, but also in other colours). Therefore, these features are termed *selection features.* Thus, the independent manipulation of the two additional selection features (location and colour) of the distractors gave rise to three different distractor types that varied in the extent of their feature overlap with the current top-down set. That is, either three overlapping features (e.g., two selection features: location congruent and colour congruent; and one response feature: shape compatibility), two overlapping features (e.g., one selection feature: location incongruent and colour congruent, or location congruent and colour incongruent; and one response feature: shape compatibility), or one overlapping feature (e.g., no selection feature: location incongruent and colour incongruent; and one response feature: shape compatibility) was presented in each trial.

Based on our previous research (Mast & Frings, 2014), we hypothesized that the strength of attentional capture would vary as a function of the feature overlap between the distractor and the attentional control sets (for an illustration, see Fig. 1a). When the target is always centrally presented and coloured green, the largest compatibility effects were expected for the two selection features overlapping distractors (e.g., green, centrally presented distractors). Medium-sized compatibility effects were expected for the one selection feature overlapping distractors (e.g., green *or* centrally presented distractors), and the smallest compatibility effects were expected for the no selection feature overlapping distractors (e.g., neither green nor centrally presented distractors). Please note that the *response feature* of the top-down set always overlapped with the distractor (as the latter was always presented as a circle or square), therefore compatibility effects were still expected for the no selection feature overlapping condition.

However, such experiments are burdened by the possible confound of perceptual/response priming effects (Kiesel et al., 2007; Wiggs & Martin, 1998). In order for the distractor to match the features incorporated in the top-down set as proposed in the multi-feature account, the distractor needs to match the target perceptually. Yet, a distractor that perceptually matches the subsequently presented target might just prime the target’s representation and thereby facilitate responses to the target (irrespective of any feature overlap with the attentional control set). More critically, a perceptual/response priming account would predict the same data pattern as a multi-feature attentional control set account. As feature overlap decreases, the perceptual overlap also necessarily decreases, and therefore the same data pattern, a decrease in the size of the compatibility effect, is predicted by both accounts. Therefore, perceptual feature overlap needs to be accounted for in order to interpret any findings in line with a multi-feature account.

To address any influence of perceptual overlap between distractor and target, we designed a control condition in which the attentional control set did not include the selection features, but which allowed us to assess any influence of perceptual feature overlap. Therefore, in a control condition (the *randomized target* condition), neither of the two selection features could be predicted accurately by the participants, as the target could be presented in any colour and from any location (see Fig. 1). Hence, the target was not predictable as far as these feature dimensions were concerned, and, consequently, the attentional control sets should not be compiled with these selection features. Crucially, identical target-distractor sequences can be presented in the two target conditions, allowing us to account for any influence of purely perceptual feature overlap between distractor and target. Thus, any difference observed between the fixed and the randomized target condition cannot be explained by differences in the bottom-up effects but only by differences in the participants’ expectations (the attentional control sets).

In all three experiments, shape was used as the *response* feature. Additionally, the temporal feature of the target (being the second stimulus) served as the first *selection feature*. In Experiment 1, location and colour were additionally used as the *selection features*. The two experimental contexts were manipulated on a between-participants basis. In Experiment 2, location was replaced as one of the *selection features* by orientation in order to demonstrate that the outlined predictions are not restricted to spatial manipulations. Furthermore, the two experimental conditions were manipulated on a within-participants basis. In Experiment 3, the number of additional selection features was increased to three, namely colour, orientation and location. To foreshadow the results, across three experiments we observed a decreasing influence of the distractor with decreasing feature overlap with the attentional control set, even when perceptual/response priming effects were accounted for.

## Experiment 1

In Experiment 1, colour and location were used as the selection features and shape as the response feature. When the target’s location (centrally presented) and its colour (green) were known (fixed target condition), the participants were expected to use this information to set up their attentional control sets with location and colour as the selection features. The size of the compatibility effect was expected to vary as a function of the feature overlap between the distractor and the top-down sets; a decrease in the feature overlap should result in a decrease in the size of the compatibility effect. As outlined above, a decline in the size of the compatibility effect is confounded by a decrease in perceptual priming, and consequently cannot unequivocally be taken as evidence for a multi-feature account of contingent capture. Thus, a control condition was incorporated into the experimental design in order to measure the influence of the perceptual similarity between the distractor and the target (not the top-down sets) on the size of the attentional capture effects, the randomized target condition. As for the targets, neither the target’s location nor its colour could be accurately predicted by the participants and, consequently, could not be used to set up attentional control sets. To test whether attentional control sets work over and above perceptual priming effects, the results of the fixed target condition were tested against the results of the randomized target condition. On a statistical level, a main effect of feature overlap was expected from both a perceptual priming account and the multi-feature control account. Yet, evidence in favour of binary, multi-feature control over attentional capture would be that the decrease in the size of the compatibility effects in the fixed condition is steeper than in the randomized target condition (because only in the fixed condition can the participants set up attentional control sets in order to separate the target from the distractor). In other words, an interaction between feature overlap and target condition is expected.

### Methods

#### Participants

Sixty students from the University of Trier took part in this study.^1^ However, due to a set-up error, the data of one participant was not saved. Therefore, the data of 59 students were analysed, 29 students (seven male, 22 female; mean age 21.5 years) participated in the randomized target features condition, and another 30 (seven male, 23 female; mean age 23.9 years) in the fixed target features condition. All of the participants reported normal or corrected-to-normal vision. All of the participants gave their written, active informed consent prior to taking part.

#### Stimuli and apparatus

In order to reduce the amount of background environmental noise to a minimum, the experiment was conducted in one of three soundproofed cabins. The instructions as well as the visual stimuli were presented on a 22-in. monitor (Model FlexScan S2202 W, EIZO Europe GmbH, Mönchengladbach, Germany). The monitor (60-Hz refresh rate), was placed approximately 50 cm in front of the participant’s body midline. The distance was held constant by means of a chinrest. Responses were assessed with the help of a standard PC mouse (connected via USB-port). Data were analysed with IBM SPSS (Version 26).

The stimuli used in this study were either a square or circle (1.49° visual angle). They were displayed in one of three possible colours: green (RGB-vlaue: 0, 128, 0; CIE L*a*b*-value: 46, -52, 50), red (RGB-value: 255, 0, 0; CIE L*a*b*-value: 53, 80, 67) and blue (RGB-value: 0, 0, 255; CIE L*a*b*-value: 32, 79, -108). Moreover, the location of the distractors as well as the location of the target in the randomized target condition varied throughout the experiment, being presented from the centre of the screen or else 3.72° (3.25 cm) above or below the centre. The centre of the screen was indicated by a fixation plus sign (0.4° visual angle) at the start of each trial.

#### Procedure

During each trial, two stimuli were presented successively. The participants were instructed to try to ignore the distractor as much as possible, and to respond as rapidly and accurately as possible to the identity (i.e., shape) of the visual target according to their respective stimulus-response mapping. The identity (e.g., the response feature shape) of the distractor stimulus was uninformative with regard to the identity of the subsequently presented target. Each trial started with the central presentation of the fixation plus sign for 500 ms. This first display was followed immediately by the appearance of the distractor stimulus for 30 ms. In-between the presentation of the target and the distractor display, a blank screen was presented for a variable interval (the distractor-target interstimulus interval (ISI); 50, 90 or 130 ms). Finally, the target was presented for 30 ms. The participants had to respond to the shape of the target within 1,030 ms of target onset. The stimulus response mapping was balanced across participants (left mouse response mapped to the circle and the right mouse response mapped to the square, or vice versa). After a response had been detected, the next trial started following a 300-ms blank interval.

In the response compatible trials, the targets had the same shape as the distractor. By contrast, in the response incompatible trials, the target and the distractor had different shapes. In order to manipulate the feature overlap between the targets and the distractors, the colour and location of the distractor were manipulated as a function of the subsequent target. Thus, four different types of trials were assigned to the three feature overlap conditions as follows: When the distractor matched the response feature as well as both properties of the subsequent target (congruent colour *and* congruent location), those trials were assigned to the three features overlapping condition (3 FO). Those trials in which the distractor only matched the target’s shape (incongruent colour *and* incongruent location) were assigned to the one feature overlapping condition (1 FO). Finally, those trials in which the distractor matched the response feature and one of the target’s selection features (either congruent colour and incongruent location, or incongruent colour and congruent location) were assigned to the two feature overlapping condition (2 FO). All of the four different trial types were equally likely (25%) but varied randomly on a trial-by-trial basis.

Two separate experimental blocks were designed for the two target conditions; the fixed target condition (the experimental condition) and the randomized target condition (the control condition). In Experiment 1, each participant only worked through one of the experimental blocks, in the subsequent two experiments, both experimental blocks were experienced by all participants. In the fixed condition, the target was always presented in the same colour (green) and from the same location (the centre of the screen). Consequently, the target’s colour and location could be predicted by the participant. By contrast, in the randomized condition, neither the target’s colour nor its location was fixed. The target could be presented in one of three different colours (green, red, blue) and from one of three different locations (centrally, 3.72° above, 3.72° below the centre).

The experiment was initiated by a short practice phase (36 training trials) where feedback was provided after every response. The practice phase was followed by 576 experimental trials (2 × response compatibility × 2 location congruency × 2 colour congruency × 3 ISI × 24 repetitions – please note that the target condition was manipulated as a between-participant factor). The participants were offered a break every 40 trials and feedback was provided by the program concerning their accuracy rate up to that point. The participants were additionally asked whether they wanted a break if they made the wrong response in three consecutive trials.

#### Design/analysis

The data were analysed in a 3 × 2 factorial design, with one within-participants factor of *feature overlap* (three features overlapping (3 FO) vs. two features overlapping (2 FO) vs. one feature overlapping (1 FO) and one between-participants factor of *target condition* (fixed vs. randomized). To arrive at this design, the location congruency as well as colour congruency factor were combined to result in the three feature overlap conditions of 1 FO (colour incongruent and location incongruent), 2 FO (colour congruent and location incongruent as well as colour incongruent and location congruent), and 3 FO (colour congruent and location congruent). Additionally, the ISI factor was dropped for analysis, as this factor was only manipulated to prevent participant’s allocation of their attention to a specific point in time (see Nobre, 2001; Nobre & Coull, 2010). Furthermore, previous experiments have shown that ISI typically only shows a main effect, but no modulation of feature overlap.^2^ The participants were randomly assigned to the two target conditions. All of the analyses were computed with compatibility effects (i.e., the difference between response compatible and response incompatible trials) as the dependent variable, for reaction times (RTs) as well as error rates.

### Results

Only those trials in which the participant responded correctly to the target were considered for the analysis of RTs. Additionally, all of those trials with an RT that was three interquartile ranges above the third quartile of each participant’s individual RT distribution were excluded from the data analyses (Tukey, 1977). Of all trials, 7.9% were excluded from the analysis due to these restrictions. See Table 1 for the mean RTs and error rates. As the dependent variable, compatibility scores were calculated by dividing the mean value for incompatible trials by the mean values for compatible trials for each participant. For violations of sphericity, Greenhouse-Geisser corrections were used. The data for all experiments are openly accessible (see Open Practices Statement).

**Table 1:** Mean reaction times (RTs; in ms) and mean error rates (in brackets; in percentages) as a function of the response compatibility (incompatible vs. compatible), target features (fixed vs. randomized) and feature overlap (3, 2 or 1 feature overlap) in Experiment 1. Compatibility effects reflect the difference between response incompatible and response compatible trials

Target condition	Fixed				Randomized			
	3 features overlap	2 features overlap		1 feature overlaps	3 features overlap	2 features overlap		1 feature overlaps
Selection feature l: Color	*Congruent*	*Congruent*	*Incongruent*	*Incongruent*	*Congruent*	*Congruent*	*Incongruent*	*Incongruent*
Selection feature ll: Location	*Congruent*	*Incongruent*	*Congruent*	*Incongruent*	*Congruent*	*Incongruent*	*Congruent*	*Incongruent*
Incompatible	546 (14.3)	521 (10.1)	530 (10.4)	498 (7.0)	584 (14.9)	577 (12.0)	584 (14.0)	569 (12.2)
Compatible	417 (3.1)	435 (3.5)	442 (3.4)	446 (3.8)	447 (3.1)	484 (3.4)	456 (3.3)	479 (3.4)
Compatibility effect	128 (11.2)	86 (6.6)	87 (7.0)	52 (3.2)	137 (11.9)	93 (8.6)	129 (10.8)	90 (8.9)

#### Reaction times (RTs)

**Fig. 2:**
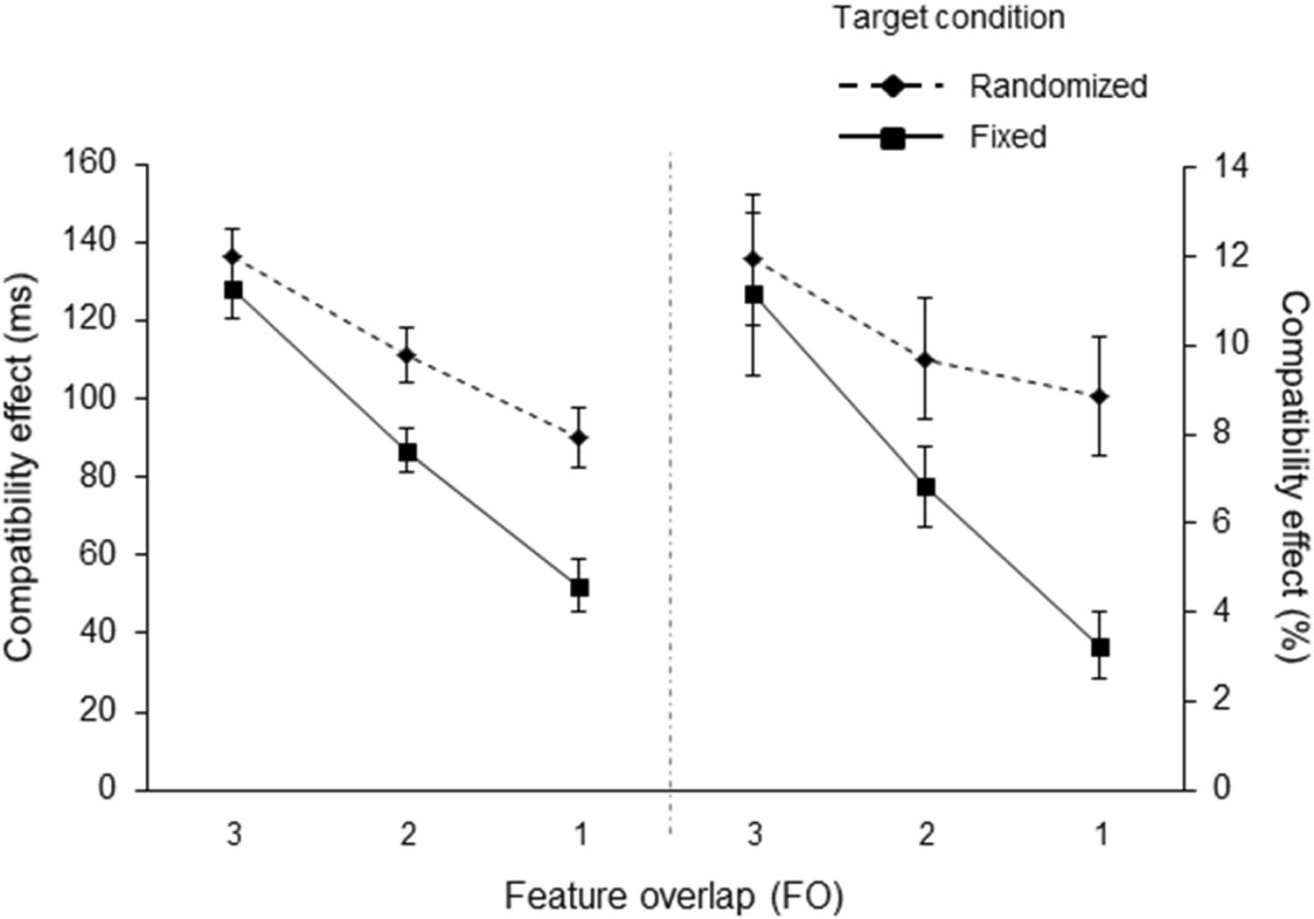
Compatibility effects for reaction time (in ms; on the left) and error rate (as percentages; on the right) in Experiment 1 as a function of feature overlap (3, 2, vs. 1) and target condition (fixed vs. randomized). Compatibility effects were computed as the difference between response incompatible and response compatible trials. The error bars depict the standard errors of the means

A 3 (feature overlap: 3 FO vs. 2 FO vs. 1 FO) × 2 (target features: fixed vs. randomized) ANOVA (analysis of variance) was conducted with compatibility effects as the dependent variables. The ANOVA revealed a significant main effect of the target feature, *F*(1,57) = 7.688, *p* = .007, η_p_^2^ = .119, with larger compatibility effects being reported for the randomized target condition (mean size of the compatibility effects: 112 ms) than for the fixed target condition (mean size of the compatibility effects: 89 ms). The main effect of feature overlap was significant, *F*(1.58, 90.13) = 96.389, *p* < .001, η_p_^2^ = .628, highlighting a decreasing compatibility effect with decreasing feature overlap as shown by the significant linear trend test, *F*(1,57) = 129.554, *p* < .001, η_p_^2^ = .694. Crucially, in terms of the present study, the ANOVA revealed a significant interaction between feature overlap and target condition, *F*(1.58, 90.13) = 3.836, *p* = .008, η_p_^2^ = .091 (see also Fig. 2), thus showing that the decrease in the magnitude of the compatibility effect was modulated by the predictability of the target’s features. According to our main hypothesis, we further examined this interaction. In fact, the interaction reflected significantly different linear trends, *F*(1,57) = 7.677, *p* = .008, η_p_^2^ = .119. That is, the decrease in the size of the compatibility effect varied as a function of the target condition. The slope is steeper in the fixed target condition (3 FO – 128 ms, 2 FO – 87 ms, 1 FO – 52 ms) than in the randomized target condition (3 FO – 137 ms, 2 FO – 111 ms, 1 FO – 90 ms).

#### Error rates (ERs)

A 3 (feature overlap: 3 FO vs. 2 FO vs. 1 FO) × 2 (target features: fixed vs. randomized) ANOVA was conducted. Comparable to the RT data, the size of the compatibility effect decreased with decreasing feature overlap, resulting in a significant main effect of feature overlap, *F*(1.42, 80.80) = 15.920, *p* < .001, η_p_^2^ = .218, with a significant linear trend, *F*(1,57) = 20.778, *p* < .001, η_p_^2^ = .267. Furthermore, the interaction of feature overlap and target condition was significant, *F*(1.42, 80.80) = 2.839, *p* = .034 (one-tailed), η_p_^2^ = .051 (see also Fig. 2). In line with the RT analyses, the linear trend for this interaction was significant, *F*(2,56) = 4.055, *p* = .049, η_p_^2^ = .066. That is, the decrease in the size of the compatibility effects was steeper in the fixed target condition (3 FO – 11.12%, 2 FO – 6.83%, 1 FO – 3.24%) than in the randomized target condition (3 FO – 11.93%, 2 FO – 9.70%, 1 FO – 8.86%). The main effect of target features, with fewer errors in the fixed condition (7.08% to 10.16%), also reached statistical significance, *F*(1,57) = 4.294, *p* = .043, η_p_^2^ = .070.

### Discussion

In Experiment 1, multiple distractor features were manipulated in order to examine whether participants set up their attentional control sets for more than one selection feature at a given time. In the fixed target condition, the targets were always presented from the same central location and were always green. By contrast, the distractors were randomly presented from any one of the three possible locations (centrally, above, or below the centre) and in any one of three possible colours (green, red or blue). Thus, colour and location could be used to separate the targets from the distractors (at least in 75% of all trials). In line with our hypotheses, the size of the compatibility effects decreased as a function of the feature overlap between the distractor and the target (see Fig. 1 for a graphical explanation). The largest compatibility effects were obtained when the distractor matched the colour and the location of the subsequent target (centrally presented, green distractors). Medium-sized compatibility effects were obtained when the distractors matched either colour *or* location (either green or centrally presented distractors), and the smallest compatibility effects were documented when the distractors neither matched in terms of their colour nor in terms of their location (i.e., distractors presented in red or blue and from above or below the centre).

The pattern of results that was obtained for the fixed target condition can also be explained by a weaker priming effect as the perceptual feature overlap decreases identically with the decrease of the feature overlap with the attention control set (3 vs. 2 vs. 1 feature overlap, see Fig. 1). Research on perceptual priming (see Wiggs & Martin, 1998) and repetition priming (see Grill-Spector et al., 2006) suggests that as the physical similarity between the prime (here, the distractor) and the target decreases the strength of perceptual priming effects. Thus, a perceptual priming account would lead to the same predictions for the fixed target condition in the present study as would the contingent capture account. To address this issue, an additional randomized target condition was used. In the randomized target condition, the participants could not foresee the target’s colour nor the location where it would be presented from. Thus, these features could not be used as selection features to separate the target from the distractors. Consequently, the participants could not compile their attentional control sets with colour and location as features. Therefore, with decreasing perceptual feature overlap between the distractor and target, a decrease in the compatibility effect is predicted by classical repetition priming/perceptual priming accounts (e.g., Wiggs & Martin, 1998). Yet, critically, this decrease should be stronger for the condition in which not just the perceptual feature overlap, but also the feature overlap between the distractor and the attentional control set is changing (fixed target condition). This is exactly what we observed. The decline in the size of the compatibility effects was more pronounced in the fixed target condition than in the randomized target condition. This is an important observation because it rules out the possibility that the results observed in the fixed target condition could be explained exclusively by differences in the perceptual overlap between the distractor and the subsequent target irrespective of top-down mechanisms. Even though top-down sets are not the only factor when it comes to explaining the results of Experiment 1, they nevertheless play a major role beyond perceptual priming effects.

One might question the fact that location was chosen as a possible selection feature, given that it has been claimed that, as a feature, location has a unique role as compared to other features such as colour, orientation or shape (Tsal & Lavie, 1993; see also Fitousi, 2016). This becomes especially important when comparing the results of the fixed target condition with those of the randomized target condition. In the fixed target condition, the participants should aim to focus their spatial attention on only a small central location where they knew that the target would definitely be presented from. According to the classic zoom lens metaphor (e.g., Eriksen & St James, 1986; LaBerge, 1983), participants are supposed to narrow their ‘attentional spotlight’. A stimulus that is presented outside the attentional spotlight (the peripheral distractors) will then receive less processing resources than those distractors presented from the attended location (central distractors). By contrast, in the randomized target condition, the target could be presented from any one of the three locations, which should result in an enlarged ‘attentional spotlight’ as compared to the fixed target condition. Hence, differences in the size of the attended regions between the randomized and the fixed target condition might have affected the results of the present study. To rule out this potential confound, Experiment 2 was conducted with the target and distractor always presented from the same central location. Consequently, location was replaced by orientation as a selection feature (for a visualisation, see Fig. 2). However, just as in Experiment 1, shape was used as the response feature and colour as the other selection feature. Once again, two blocks of trials were presented; the fixed target condition (green, horizontal inner orientation) versus the randomized target condition (three different colours and three different inner orientations randomly intermixed). The same pattern of results should be observed as in the previous experiment; that is, a more pronounced decline in the size of the compatibility effects in the fixed target condition than in the randomized target condition.

## Experiment 2

### Method

#### Participants

Thirty students (six male, 24 female; mean age 22 years) took part in the second study. All of the participants reported normal or corrected-to-normal vision. All of the participants gave written, active informed consent prior to their taking part.

#### Design

Once again, the participants were tested in a 3 (feature overlap: 3 FO vs. 2 FO vs. 1 FO) × 2 (target condition: fixed vs. randomized) factorial design. However, in contrast to Experiment 1, the target condition was manipulated as a within-participants factor.

#### Apparatus and materials

**Fig. 3:**
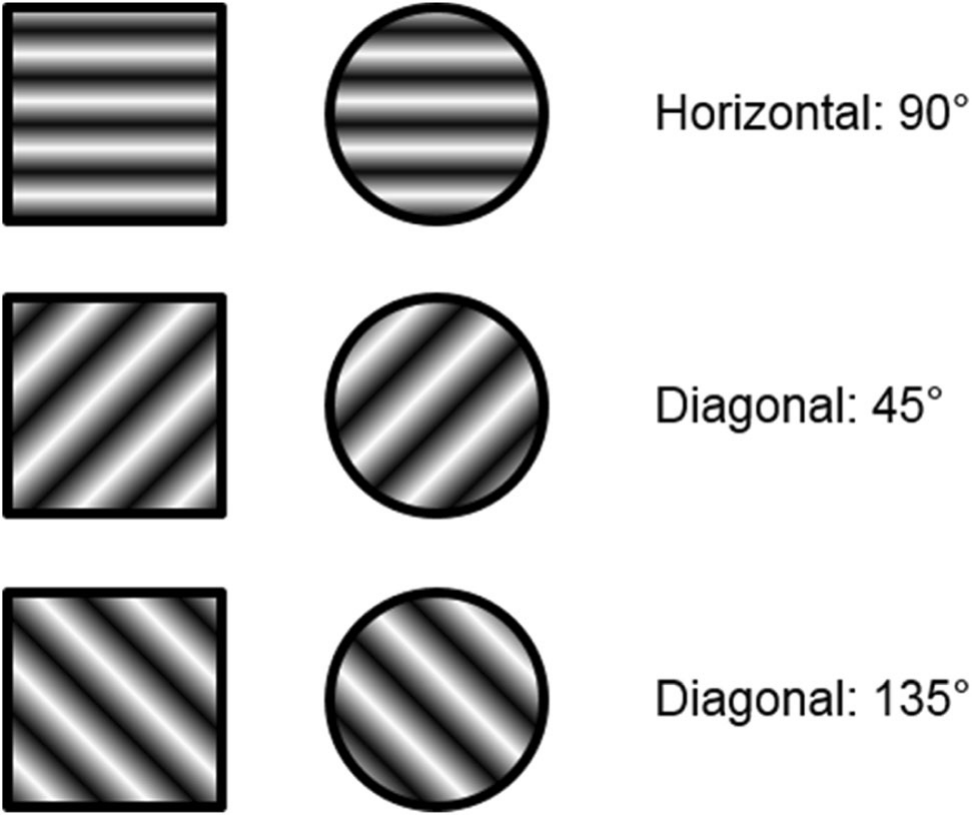
The six stimuli of different orientation (selection feature) and shape (response feature) presented in Experiment 2. The lines presented in black in this figure were coloured (green, red or blue) in the experiment

The apparatus was exactly the same as in the previous experiment. Most importantly in this experiment, all of the stimuli were presented from the same, central screen location. Black lines within the stimuli depicted the new selection feature, inner orientation (see Fig. 3 for an illustration). Those lines across the coloured stimuli were presented horizontally (90°) or diagonally at 45° or 135°. The size of the stimuli was increased slightly (2.46°). The stimuli were presented in one of three colours, which were adapted slightly to increase contrast between the black inner lines and background; green (RGB-value: 0, 204, 0; CIE L*a*b*-value: 72, -73, 70), red (RGB-value: 255, 0, 0; CIE L*a*b*-value: 53, 80, 67) or blue (RGB-value: 17, 123, 251; CIE L*a*b*-value: 53, 21, -71).

#### Procedure

The sequence of events for each trial was identical to that used in Experiment 1. In Experiment 2, target condition was manipulated as a within-participants factor. Therefore, two subsequent blocks of trials were presented (fixed targets vs. randomized targets). Within the fixed target block, the target was always presented in red and with black horizontal lines (inner orientation). By contrast, in the randomized target block, the colour and the orientation of the inner lines of the target were not predictable (see Fig. 3 for a visualization of all possible inner orientations). The sequence of two target conditions was balanced across participants. Moreover, the number of trials for each training and experimental session was adjusted to 48 training trials and 384 experimental trials. Thus, the participants performed 96 experimental trials of each trial type per target features condition. No further changes were made.

### Results

The same criteria for data exclusion were implemented as in Experiment 1. Therefore, 6.7% of all trials were excluded from the analysis due to these restrictions. Mean RTs and ERs are shown in Table 2. Once again, the size of the compatibility effects (the difference between response compatible and response incompatible trials) was used as the dependent variable.

**Table 2:** Mean reaction times (in ms) and mean error rates (in brackets; in percentages) as a function of the response compatibility (incompatible vs. compatible), target features (fixed vs. randomized), and feature overlap (3, 2, vs. 1 feature overlap) in Experiment 2. Compatibility effects reflect the difference between response incompatible and response compatible trials

Target condition	Fixed				Randomized			
	3 features overlap	2 features overlap		1 feature overlaps	3 features overlap	2 features overlap		1 feature overlaps
Selection feature l: Color	*Congruent*	*Congruent*	*Incongruent*	*Incongruent*	*Congruent*	*Congruent*	*Incongruent*	*Incongruent*
Selection feature II: Inner Orientation	*Congruent*	*Incongruent*	*Congruent*	*Incongruent*	*Congruent*	*Incongruent*	*Congruent*	*Incongruent*
Incompatible	523 (10.6)	518 (10.1)	518 (10.0)	512 (9.0)	516 (11.4)	524 (9.6)	517 (11.2)	521 (10.0)
Compatible	411 (2.4)	440 (3.5)	445 (2.7)	451 (2.7)	409 (2.6)	477 (2,8)	416 (2.7)	442 (3.0)
Compatibility effect	113 (8.2)	77 (4.3)	93 (7.3)	61 (5.2)	107 (8.8)	77 (6.8)	101 (8.5)	79 (7.0)

#### RTs

**Fig. 4:**
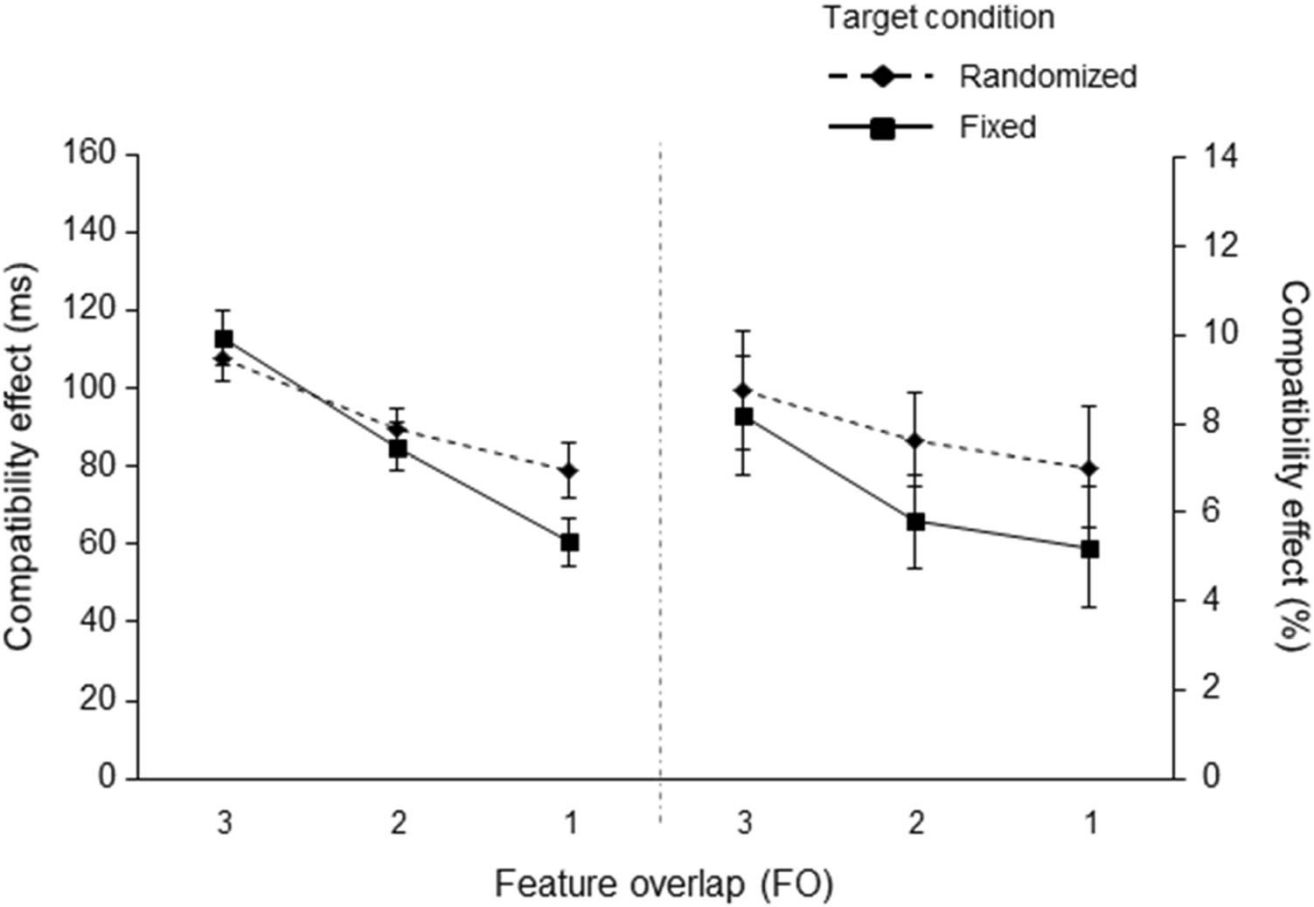
Compatibility effects for reaction times (in ms; on the left) and error rates (as percentages; on the right) in Experiment 2 as a function of feature overlap (3, 2, vs. 1) and target condition (fixed vs. randomized). Compatibility effects were computed as the difference between response incompatible and response compatible trials. The error bars depict the standard errors of the means

A 3 (feature overlap: 3 FO vs. 2 FO vs. 1 FO) × 2 (target condition: fixed vs. randomized) ANOVA was conducted. The main effect of feature overlap was significant, *F*(1.64, 47.60) = 48.26, *p* < .001, η_p_^2^ = .625, reflecting a decreasing compatibility effect with decreasing feature overlap, as shown by the significant linear trend test, *F*(1, 29) = 65.593, *p* < .001, η_p_^2^ = .693. Most crucially, however, the ANOVA revealed a significant interaction of feature overlap and target features, *F*(2,58) = 4.919, *p* = .011, η_p_^2^ = .145 (see Fig. 4). The interaction shows that the decrease in the size of the compatibility effect was modulated by the target condition. As in Experiment 1, the linear trend of this interaction reached significance, *F*(1,29) = 8.081, *p* = .008, η_p_^2^ = .218. This result reveals that the decrease in the magnitude of the compatibility effects was more pronounced in the fixed condition (3 FO – 113 ms to 2 FO – 85 ms to 1 FO – 61 ms) than in the randomized condition (3 FO – 107 ms to 2 FO – 89 ms to 1 FO – 79 ms). The main effect of target condition was not significant, *F*(1,29) = 2.688, *p* = .112, η_p_^2^ = .085.

#### ERs

A 3 (feature overlap: 3 FO vs. 2 FO vs. 1 FO) × 2 (target condition: fixed vs. randomized) ANOVA was conducted. The main effect of feature overlap was significant, *F*(2,58) = 4.732, *p* = .012, η_p_^2^ = .140. Once again, the significant linear trend test, *F*(1,29) = 8.249, *p* = .008, η_p_^2^ = .221, indicates decreasing compatibility effects with decreasing feature overlap (3 FO – 8.42% to 2 FO – 6.72% to 1 FO – 6.11%). The interaction term, although descriptively comparable to the RT data (see Fig. 4), was not significant, *F*(2,58) = 0.407, *p* = .667, η_p_^2^ = .014, as well as the main effect of target features, *F*(1,29) = 1.992, *p* = .176, η_p_^2^ = .062.

### Discussion

The primary aim of Experiment 2 was to rule out the possibility that location (as a selection feature) might have given rise to differences in the size of the attended area between the randomized and the fixed target condition in Experiment 1. Such a confound might have been expected to systematically influence distractor processing in the randomized and in the fixed target conditions. In order to rule out this potential confound, location was replaced by stimulus orientation as a selection feature. Hence, the distractors and the targets were always presented from the same central location, irrespective of the target condition. The results of Experiment 2 mirror those seen in Experiment 1. In both conditions, as the featural overlap decreased, the size of the compatibility effects decreased as well. However, in the fixed target condition, this decrease in the size of the compatibility effects was more pronounced as compared to the randomized target condition. These results are therefore in line with the idea that participants can set up their own attentional control sets for multiple selection features. As the feature overlap between the distractor and the top-down sets decreases, the distractors’ potential to capture attention decreases as well. Again, a decrease in the perceptual priming condition was examined (randomized target condition). But, as in the previous experiment, the decrease in perceptual priming (as observed in the randomized target condition of Experiment 2) cannot account for the entire pattern of results in the fixed target condition where both top-down sets and perceptual priming effects seems to modulate the decrease in size of the compatibility effect.

The results of Experiment 2 are in line with a multi-feature extension of contingent capture. Thus, differences in the size of the area that is attended can be ruled out as a confounding factor in our methodological design. The aim of Experiment 3 was to further stress the predictions raised by multi-feature attentional control sets. Hence, three instead of two selection features were manipulated. That is, the colour, orientation and location of the distractors could differ from the participants’ top-down sets. To rule out the possibility that location incongruent trials are mainly responsible for the expected differences, the location congruent trials were analysed separately. Once again, we expected to observe a monotonous decrease in the size of the compatibility effects.

## Experiment 3

### Method

#### Participants

Thirty students (eight male, 22 female; mean age 22 years) took part. All of the participants reported normal or corrected-to-normal vision. All of the participants gave written, active informed consent prior to participation.

#### Design

The design was adjusted and the participants were tested in a 4 (feature overlap: 4 FO vs. 3 FO vs. 2 FO vs. 1 FO) × 2 (target condition: fixed vs. randomized) factorial design. All of the factors were manipulated within-participants.

#### Apparatus and materials

The apparatus and materials were exactly the same as in Experiment 2. The location of the stimuli varied in a manner that was identical to Experiment 1.

#### Procedure

The sequence of events for each trial was identical to the procedure in Experiment 2. In the fixed target feature condition, the target was always presented from the centre of the screen, with one fixed colour (red) and a fixed orientation of the inner lines (horizontal). In the randomized target condition, the target was not predictable in terms of its location, colour or inner orientation. Moreover, the number of trials in each training and experimental session was adjusted to 48 training trials and 288 experimental trials. Thus, the participants executed 72 experimental trials in each of the four feature overlap conditions. Hereby, the type of trial was selected randomly in each of the four conditions. No further changes were made.

### Results

The same rules were used as in the previous experiments leading to 7.3% of all trials being excluded from further analysis. Mean RTs and ERs are shown in Table 3. The size of the compatibility effects (the difference between response compatible and response incompatible trials) was used as the dependent variable.

**Table 3:** Mean reaction times (in ms) and mean error rates (in brackets; in percentages) as a function of the response compatibility (incompatible vs. compatible), target features (fixed vs. randomized) and feature overlap (4, 3, 2 or 1 feature overlap) in Experiment 3. Compatibility effects reflect the difference between response incompatible and response compatible trials

Target condition	Fixed				Randomized			
	4 features overlap	3 features overlap	2 features overlap	1 feature overlaps	4 features overlap	3 features overlap	2 features overlap	1 feature overlaps
Incompatible	527 (7.0)	517 (5.6)	509 (3.8)	466 (2.1)	554 (6.6)	555 (6.8)	560 (6.7)	561 (6.7)
Compatible	402 (1.0)	427 (1.1)	434 (1.4)	446 (0.9)	436 (2.3)	456 (2.3)	458 (1.5)	472 (1.3)
Compatibility effect	125 (6.0)	90 (4.4)	75 (2.4)	20 (1.2)	119 (4.4)	119 (4.8)	92 (5.1)	89 (5.4)

#### RTs

**Fig. 5:**
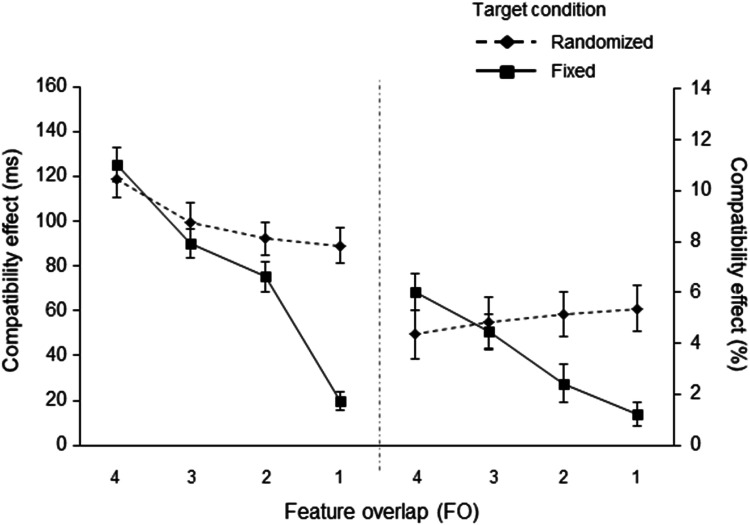
Compatibility effects for reaction time (in ms; on the left) and error rate (as percentages; on the right) in Experiment 3, as a function of feature overlap (4, 3, 2, vs. 1) and target condition (fixed vs. randomized). Compatibility effects were computed as the difference between response incompatible and response compatible trials. The error bars depict the standard errors of the means

A 4 (feature overlap: 4 FO vs. 3 FO vs. 2 FO vs. 1 FO) × 2 (target condition: fixed vs. randomized) ANOVA was conducted. As in the previous experiments, a main effect of the feature overlap factor was observed, *F*(2.37, 68.68) = 61.242, *p* < .001, η_p_^2^ = .679. The main effect was further qualified by a linear trend test, which again reached significance, *F*(1,29) = 107.854, *p* < .001, η_p_^2^ = .788. The size of the compatibility effect decreased as the feature overlap decreased. The ANOVA revealed a significant interaction between the feature overlap and the target condition, *F*(3,87) = 24.832, *p* < .001, η_p_^2^ = .461 (see also Fig. 5). As in the previous experiments, a significant linear trend test of this interaction, *F*(1,29) = 61.113, *p* < .001, η_p_^2^ = .678, was observed. This shows that the decrease in the size of compatibility effects over the different feature overlap conditions was more pronounced in the fixed target condition (4 FO – 125 ms; 3 FO – 90 ms; 2 FO – 75 ms; and 1 FO – 20 ms) than in the randomized target condition (4 FO – 119 ms; 3 FO – 100 ms; 2 FO – 92 ms; and 1 FO – 89 ms). The main effect of target condition was also significant, *F*(1,29) = 43.517, *p* < .001, η_p_^2^ = .600, with an overall smaller compatibility effect in the fixed target condition (78 ms as compared to 100 ms in the randomized target condition).

Additionally, we examined only the spatially congruent trials to underpin the results from Experiment 2 and to rule out the possibility that the observed decrease in the size of the compatibility effect as a function of the feature overlap is mainly driven by the spatial feature. Therefore, a 3 (feature overlap: 3, 2, vs. 1 FO) × 2 (target condition: fixed vs. randomized) ANOVA was conducted for only the spatially congruent trials. Once again, the size of the compatibility effects decreased with decreasing feature overlap as indicated by the significant main effect for feature overlap, *F*(2,58) = 28.025, *p* < .001, η_p_^2^ = .491. In line with the results of Experiment 2, the interaction of feature overlap and target condition was significant, *F*(2,58) = 6.326, *p* = .003, η_p_^2^ = .179. Once again, the linear trend test for this interaction reached significance, *F*(1,29) = 11.883, *p* = .002, η_p_^2^ = .291. That is, the decrease of the compatibility effects was more pronounced in the fixed condition (3 FO – 125 ms; 2 FO – 84 ms; 1 FO – 62 ms) than in the randomized target condition (3 FO – 119 ms; 2 FO – 101 ms; 1 FO – 95 ms). Furthermore, a main effect for the target condition was observed, *F*(1,29) = 6.871, *p* = .014, η_p_^2^ = .192, with larger compatibility effects in the randomized target condition than in the fixed target condition.

#### ERs

A 4 (feature overlap: 4, 3, 2, vs. 1 FO) × 2 (target condition: fixed vs. randomized) MANOVA with Pillai’s trace as criterion was conducted. Comparable to the RT analyses, the size of the compatibility effect decreased with decreasing feature overlap, resulting in a significant main effect of feature overlap, *F*(3,87) = 3.591, *p* = .017, η_p_^2^ = .110, with a significant linear trend test, *F*(1,29) = 10.008, *p* = .004, η_p_^2^ = .257. Furthermore, the interaction of feature overlap and target condition was significant, *F*(3,87) = 8.879, *p* < .001, η_p_^2^ = .234 (see Fig. 5). As for the RTs, the linear trend test for the interaction was also significant, *F*(1,29) = 17.438, *p* < .001, η_p_^2^ = .376. That is, the compatibility effects decreased in the fixed condition (4 FO – 6.01%; 3 FO – 4.44%; 2 FO – 2.41%; 1 FO – 1.20%), but not in the randomized condition (4 FO – 4.35%; 3 FO – 4.82%; 2 FO – 5.13%; to 1 FO – 5.37%). Additionally, the main effect of target condition led to significant results, *F*(1,29) = 7.116, *p* = .012, η_p_^2^ = .197, with fewer errors in the fixed target condition (3.51%) than in the randomized target condition (4.92%).

As for the RT analysis, the error rates of only the spatially congruent trails were examined with a 3 (feature overlap: 3, 2, vs. 1 FO) × 2 (target condition: fixed vs. randomized) ANOVA. The main effect of feature overlap did not reach significance, *F*(2,58) = 1.815, *p* = .172, η_p_^2^ = .059. Yet, importantly, the interaction of feature overlap and target condition reached significance, *F*(2,58) = 8.100, *p* = .001, η_p_^2^ = .218, as did the linear trend test, *F*(1,29) = 15.727, *p* < .001, η_p_^2^ = .352. Thus, the decrease in the size of the compatibility effects was more pronounced in the fixed condition (3 FO – 12.04%; 2 FO – 10.13%; 1 FO – 4.44%) than in the randomized condition (3 FO – 8.7%; 2 FO – 10.56%; 1 FO – 11.67%).

### Discussion

The main aim of Experiment 3 was to test whether the impact of the distractor could be further reduced when the feature overlap was varied across three (colour, inner orientation and location), rather than two, selection features. We would argue once again that the results provide robust evidence for multi-feature attentional control sets. A dramatic drop in the size of the compatibility effects across the different feature overlap conditions was observed, but only in the fixed target condition (dropping from 125 to 20 ms) and not in the randomized target condition (from 119 to 89 ms). However, even though distractors in the one FO condition (fixed target condition) differed from the targets in terms of their colour, their inner orientation and their presentation from a location where the participants knew that the target would never be presented, attentional capture effects were nevertheless still obtained (*M*_0 FO_ = 20 ms, *t*(29) = 4.769, *p* < .001). This is presumably attributable to the fact that the distractors were always presented in one of the two shapes that were mapped onto one of the two opposing alternative responses. Thus, the distractors matched the response feature (shape) of the top-down sets and might therefore still capture the participants’ attention. Even though the 1 FO distractors differed significantly from the targets in terms of the three selection features, there was still the response feature shared by the targets and the distractors. As for Experiments 1 and 2, differences in the strength of perceptual priming effects (as measured in the randomized target condition) cannot account for the results observed in the fixed target condition. Furthermore, the results cannot be ascribed to the manipulation of location as one of the selection features. The analysis of only spatially congruent trials revealed exactly the same pattern of results as in Experiment 2.

## General discussion

The interplay of top-down and bottom-up mechanisms in the control of selective attention has been a topic of lively debate (for reviews, see Awh et al., 2012; Burnham, 2007). According to the contingent capture hypothesis (Folk et al., 1992), a stimulus (i.e., a distractor or cue) has to match the current attentional control settings in order to involuntarily capture spatial attention. To date, research on the topic of contingent capture has focused primarily on manipulations of a single feature separating the target from the distractors (see Kiss et al., 2013, for an exception) in exogenous, spatial cuing tasks (but see also Folk et al., 2008, and Mast & Frings, 2014; Mast et al., 2015, for non-spatial tasks). According to the contingent capture hypothesis, attentional control sets were thought of as a search template for a specific physical feature (e.g., colour, size, motion). Those stimuli (distractors or targets) that match participant’s current attentional control sets are selected automatically. The present study was designed to examine whether participants can set up their attentional control sets for multiple features (e.g., colour *and* orientation) at the same time. In line with a binary multi-feature account of contingent capture (see Mast & Frings, 2014), the size of attentional capture effects is assumed to be determined by the feature overlap between the features of the current distractor and the participant’s current attentional control sets. A response compatibility task was used in order to test for attentional control sets containing multiple features.^3^ During each trial, two stimuli were presented in close temporal proximity. These stimuli were either mapped on to the same or opposing responses (compatible vs. incompatible trials, respectively). Even though the identity of the distractor stimulus was not correlated with the identity of the subsequent target, strong interference effects (incompatible trials minus compatible trials) were observed. Intriguingly, in all of the experiments reported here, a decrease in the magnitude of the compatibility effects was found as the feature overlap between the distractors and the participants’ attentional control sets decreased. To interpret the observed pattern of results in favour of contingent capture, perceptual priming as a plausible alternative hypothesis had to be controlled for (fixed vs. randomized target condition). Strikingly, compared to a randomized target condition where the participants could not set up attentional control sets for additional selection features, a more pronounced decrease of the distractor’s influence was observed when the participants were able to foresee the target’s features (fixed target condition). However, one might challenge the fact that across all experiments, even in the 1 FO condition, the distractors could not be completely ignored by the participants (reliable compatibility effects in the 1 FO conditions). The presence of distractor interference effects in all of the conditions is likely attributable to the match of the response features. In contrast to typical contingent capture tasks (the exogenous cuing task), the distractor always matches the response-relevant feature of the top-down sets.

The present study provides a new perspective from which to consider the data from earlier studies on contingent capture. Take, for instance, the original study from Folk et al. (1992). There, the participants had to classify the shape of a target stimulus (‘X’ vs. ‘=’). The target was either a colour singleton or an onset singleton. It has been argued that participants set up their attentional control set either for the specific target colour *or* the abrupt onset. The cue that was presented briefly before the onset of the target either matched the current attentional control set or not, and only in the matching condition was attentional capture observed. This pattern of results was interpreted in favour of a top-down set guiding attention according to a task-relevant target feature. However, according to a multi-feature perspective on contingent capture, the participants should have implemented at least one additional selection feature into their top-downs sets, that is, shape. In Folk et al.’s study, the cues were presented as four dots surrounding one of the potential target locations. However, as mentioned above, the targets were either ‘X’s or ‘=’s. Thus, shape could have been used by the participants in order to separate the target from the cues (distractors). As indicated by the results of the present study, it is more difficult to ignore a distractor that matches two features of the current top-down sets than a distractor that matches only one feature of the top-down sets. Note that this conclusion does not counter the classic contingent capture hypothesis but rather enables more precise predictions concerning the occurrence and the strength of attentional capture effects. Accordingly, the results presented here show that in research on contingent capture it is important to be sensitive to all of the features that vary in a given task. That is because they may be used by the participants to separate the target from the distractors, regardless of whether the distractors are presented at the target display or briefly before the target’s onset (the cues).

An interesting aspect here is the linear decrease in the attentional capture effects as a function of the feature overlap. Thus, a distractor does not have to match the entire feature conjunction to become automatically selected (e.g., see Schäfer et al., 2016, for a study examining single feature and conjunction processing). Instead, the strength of attentional capture effects has been shown to linearly decrease as the feature overlap between the distractor and the current top-down sets decreases. These observations are in line with studies from Ansorge and Heumann (2003, 2004) in which target-distractor similarity was manipulated. In their studies, the participants had to respond to a target of a specific colour (e.g., blue). Intriguingly, more pronounced attentional capture effects have been documented after the presentation of a target-similar distractor (e.g., a bluish-green cue) than after the presentation of a target-dissimilar distractor (yellowish-red). The results of the present study underpin the importance of target-distractor similarity as a crucial factor modulating the strength of attentional capture effects (for similar results, see also Biderman et al., 2017). In contrast to the studies reported by Ansorge and Heumann, we manipulated the target-distractor similarity across multiple features and not as a gradual change within a specific feature. Thus, the term *feature overlap* instead of *target-distractor similarity* was used in the present study, though the underlying neural mechanism might be similar (for similar ideas, see Geng et al., 2017; Yu & Geng, 2019).

The present data could also be linked to prominent theories of visual attention (e.g., Feature Integration Theory, Treisman, 1988; Treisman & Gelade, 1980, for reviews, see Quinlan, 2003; Spence & Frings, 2020; the Guided Search model, Wolfe, 1994, 2007; the Theory of Visual Attention, Bundesen, 1990; see also Desimone & Duncan, 1995, for a neural model of visual attention). According to most models of visual attention, selection is determined both by stimulus-driven (bottom-up) and goal-directed (top-down) factors. The bottom-up potential of a stimulus to become selected depends of its salience; the difference of one object as compared to the surrounding objects. A claim that is often made, when it comes to describing the impact of top-down factors, is that participants can adjust the attentional weights of specific features due to the current task requirements (e.g., when searching for a green target, green objects become prioritized). Intriguingly, the impact of different features can be adjusted independently for multiple features at a time. The multi-feature account of contingent capture is in line with these models of visual attention. The participants may have adjusted the attentional weights for those features that were incorporated into their top-down sets. Thus, the compilation of top-down sets with multiple features closes an important theoretical gap when it comes to our understanding of visual attention on a more general level.

The present results are also neatly in line with current theoretical developments in the action control literature. While earlier accounts have already described important influence of top-down control states like action-triggers (Kunde et al., 2003) or as a sort of prepared reflex (Hommel, 2000), recently, binding and retrieval as two separate processes have been described as core components for action control (Frings et al., 2020). Hereby, attentional control setting processes have been proposed to influence the to-be-compiled event files (Hommel, 1998, 2004), in which encountered stimulus, response and effect information are stored. The results reported here indicate that the weights of the stored feature information are likely to be influenced by the attentional control settings, which are compiled based on the task-specific conditions. Yet, future studies might want to focus on the questions whether the attentional weights are influencing the compilation/binding of the distractor event file or the retrieval of the distractor event file when the target is encountered, while accounting for the perceptual overlap effects that have been reported for distractor retrieval (cf. Schöpper et al., 2020; Singh et al., 2016).

To conclude, the results of the present study provide strong evidence for a multi-feature perspective on contingent capture, an extension of the original contingent capture hypothesis by Folk and his colleagues (Folk et al., 1992). The strength of attentional capture by a task-irrelevant distractor has been found to vary as a function of the assumed feature overlap between the distractor and the participants’ current top-down sets (i.e., the target-distractor similarity). This pattern of results has been taken as evidence that participants can implement different features into their attentional control sets. Therefore, the present study provides a new perspective on contingent capture. This new perspective might be of significance in those studies where attentional capture effects have been found, even though the distractors did not match the feature that was assumed to be incorporated into the participants’ top-down sets. This is because the distractor might still match another, otherwise overseen, feature that the participants implemented in their attentional control sets.

## Footnotes

1 Based on our experience with comparable studies as well as other published data (e.g., Kiss et al., 2013), we used a conservative measure for sample size calculations (f = 0.2; α = 0.05; 1-β = 0.9; number of groups = 2; number of measurements = 3; for the between-within interaction), which resulted in an overall sample size of 56 participants. This was slightly increased to account for possible drop-outs; therefore, data for 60 participants were collected.

2 A 3 (feature overlap) × 2 (target condition) × 3 (ISI) ANOVA was conducted to investigate any potential influence of ISI on the theoretically interesting interaction of feature overlap and target condition with RT compatibility effects as dependent variable. In line with previous results (e.g., Mast & Frings, 2014), a main effect of ISI was observed, *F*(2, 114) = 42.56, *p* < .001, η_p_^2^ = .427, with stronger compatibility effects for the longer ISIs (ISI 50 = 82 ms; ISI 90: 104 ms; ISI 130 = 117 ms). Yet, crucially, the interaction of feature overlap and target condition was not further modulated by ISI, *F*(4, 228) = 2.54, *p* = .064, η_p_^2^ = .038.

3 As already discussed in the *Introduction*, it is debated whether one attentional control set including multiple features is compiled (e.g., Büsel et al., 2018; Mast & Frings, 2014), or, instead, multiple attentional control sets for each individual feature are used (e.g., Irons et al., 2012; Kerzel & Witzel, 2019; Roper & Vecera, 2012). In our view, our results are better explained by the multi-feature attention control set idea, that is, a multi-feature attentional control set would likely use much less cognitive resources compared to a cognitive system jumping between the different attentional control sets for the different features. Additionally, with one selection feature (temporal order) providing a clear distinction between target and distractor in all of the trials, it would not be sensible to switch to an attentional control set that comprises a feature that can only help to distinguish between the target and distractor in 50% of the trials (which are the other selection features such as location, colour or orientation). Therefore, the multi-attentional control set hypothesis would likely not predict an additional decrease of the compatibility effect in the fixed condition as the simple use of the temporal feature could help to separate the distractor from the target in all trials.
